# Clinician Perspectives on Integrating Mobile Sensor Data Into Cancer Care: Mixed Methods Study

**DOI:** 10.2196/86412

**Published:** 2026-05-08

**Authors:** Christianna Bartel, Leeann Chen, Krina C Durica, Jennifer Fedor, Akiera Palm, Tommy Selwood, Pari Lakhia, Roby Thomas, Heidi Donovan, Carissa A Low

**Affiliations:** 1 University of Pittsburgh, 5051 Centre Avenue, Suite 5002, Pittsburgh, PA, 15213, United States, 1 412-623-5973; 2 Morehouse School of Medicine, Atlanta, GA, United States; 3 University of Pittsburgh Medical Center, Pittsburgh, PA, United States

**Keywords:** wearable devices, oncology providers, cancer, symptom management, doctor-patient communication, remote monitoring

## Abstract

**Background:**

Wearable devices are becoming more ubiquitous and are capable of capturing health-relevant information that patients may be interested in sharing with their providers. However, limited research has been conducted on oncology provider perspectives on how these data could be used to inform cancer care.

**Objective:**

The goal of this study was to understand oncology clinicians’ preferences about which data would be most clinically valuable and in what clinical scenarios, the benefits and barriers to integrating wearable device data into cancer care, and perspectives on how wearable device data could impact decision-making using 3 clinical vignettes.

**Methods:**

A total of 13 oncology care clinicians completed an online questionnaire to assess the perceived value of different types of wearable device data in different clinical scenarios and participated in semistructured interviews to gather preferences around integrating these data into clinical workflows. During the interviews, providers were also presented with 3 clinical vignettes and asked for clinical recommendations both before and after seeing the patient’s wearable device data. Descriptive statistics were calculated to summarize quantitative data from questionnaires and structured interview questions, and interviews were transcribed and coded using an iterative thematic analysis approach.

**Results:**

Survey responses indicated that providers were most interested in tracking vital sign metrics, followed by data related to falls and functioning, and then by data on sleep and activity. Clinicians thought that wearable device data might be especially useful for remotely monitoring patients at high-risk moments in their care trajectory, such as after an acute hospitalization or after starting a new outpatient treatment. Four main themes were discussed by providers in the interviews: (1) corroborating reports, (2) identifying new issues, (3) coordinating care, and (4) patient-provider communication. Although there were no statistically significant differences in clinical recommendations before and after viewing wearable device data for any vignette (all *P*>.25), all clinicians reported that the wearable device data impacted their decision-making confidence, and most rated the wearable device data as helpful.

**Conclusions:**

Oncology providers highlighted the potential clinical value of vital sign and physical functioning data from wearable devices, particularly when outpatients might be at risk for readmissions or other acute deteriorations between clinic visits. Providers noted that the objective data captured by consumer wearable devices can be helpful complements to patient and caregiver subjective reports and that this information could improve patient-provider communication and care coordination. During the interviews, most providers found wearable device data to be helpful when making decisions. While there are challenges to address on how to integrate this information into the clinical workflow and communicate alerts with patients, there is cautious enthusiasm among clinicians about how these data could inform and improve cancer care.

## Introduction

### Background

Data from wearable devices can capture objective patterns of physical activity and physiology that may be useful for remotely monitoring patients during chemotherapy and other outpatient cancer treatments [1]. Work by our team and others suggests that it is feasible to collect wearable device data from patients during cancer treatment [2-4], that patients are willing to share these data with clinicians [5], and that these data are correlated with clinical outcomes among patients with cancer [6,7]. As these devices become more ubiquitous and powerful, research is needed to understand how best to integrate these devices and data into clinical cancer care.

In previous work, noncancer physicians reported that wearable device data may improve patient-provider communication by efficiently providing objective information about patient health, informing decision-making by monitoring response to treatment and symptom progression outside of the clinic, and helping patients feel like more active participants in their care [8,9]. Physicians also noted potential barriers to the clinical implementation of wearable device monitoring, including concerns about the digital health literacy of older patients, the potential cost of devices widening digital divides, device data increasing anxiety for some patients, and increased strain on clinical workloads [10].

In the area of oncology care, several recent studies have assessed the preferences and perspectives of patients with cancer on wearable health monitors [11,12]. Barriers noted by patients with cancer included fears that wearable devices may serve as a constant reminder of their illness and concerns that technology may replace human interactions if, for example, patients grow to rely on devices to communicate their health status to clinicians and refrain from reaching out directly. Patients also reported benefits, noting that wearable devices can be motivating, can provide new insights into their health and illness, and that they viewed the wearable device as a part of their treatment relationship and wanted their cancer treatment teams to have access to their wearable device data.

### Objective

To date, limited work has focused on cancer care clinicians’ perspectives on how wearable device data could inform cancer care. The goal of this questionnaire and interview study was to understand oncology clinicians’ preferences about which data would be most clinically valuable and in what clinical scenarios, the benefits and barriers to integrating wearable device data into cancer care, and perspectives on how wearable device data could impact decision-making using 3 clinical vignettes.

## Methods

### Recruitment

Oncology care clinicians (N=13) were recruited through purposive sampling to capture different health professionals involved in providing cancer care. Providers were eligible if they were (1) working as clinical providers caring for patients receiving cancer treatment, (2) aged over 18 years, and (3) fluent in English.

### Study Design

Participants first completed an online questionnaire to collect demographic and clinical background information, assess which sensor information they would be interested in receiving and in what scenarios, and explore potential resources that might be helpful to them in integrating wearable device data into their clinics (Multimedia Appendix 1). Semistructured interview guides were developed by the research team with a focus on preferences and needs around how technology could support care management and fit into clinical workflows (Multimedia Appendix 2).

Based on a similar study in pediatric surgery [13], participants were also presented with 3 clinical vignettes using wearable data collected as part of the remote oncology symptom assessment research protocol that followed patients receiving cytotoxic chemotherapy for solid tumors [4]. Patient participants in this study wore a Fitbit Inspire device for 90 days, and the data of 3 participants who had experienced complications during the study were presented at the end of semistructured interviews to determine whether the availability of wearable device data impacted clinician decision-making. The three vignettes presented were as follows: (1) a male patient with stage IV pancreatic cancer, aged 75 years, who received chemotherapy 2 days prior and reported worsening abdominal pain, nausea, vomiting, anorexia, and weakness over the past few days; (2) a male patient with stage IV pancreatic cancer, aged 58 years, who had last received chemotherapy 10 days prior and who reported dizziness as well as intermittent fever and chills with some abdominal pain over the past 4 to 5 days; and (3) a female patient with stage IV breast cancer, aged 56 years, whose treatment plan changed from chemotherapy to targeted therapy 2 weeks prior, following an unplanned hospitalization for cellulitis, and who now reported worsening fatigue, nausea, and decreased appetite.

The 3 clinical vignettes were presented to each clinician participant in 2 formats: without and with wearable data. First, the scenario was presented without wearable data, and participants were asked to triage the patient and determine the urgency of follow-up care. Clinicians were then asked to rate their likelihood of recommending that the patient present to the emergency department (ED) immediately using a 10-point Likert scale, with 0 representing “not at all likely” and 10 representing “very likely.”

The scenario was then augmented with wearable data. Participants were shown visualizations of wearable device data (including heart rate, gait speed, and step count data collected around the time of the phone call or visit) and were asked to rate the likelihood of recommending ED presentation using the same 10-point Likert scale. They were also asked if the wearable data increased their confidence in their recommendation and to rate how helpful the wearable device data was to their understanding of the situation on a 10-point Likert scale with 0 representing “not at all helpful” and 10 representing “extremely helpful.”

Interviews lasted between 20 and 40 minutes and were conducted remotely over Zoom (Zoom Communications Inc) by members of the research team (LC and CB). Interviews were digitally recorded, transcribed, deidentified, and entered into Dovetail (Dovetail Research Pty Ltd), a qualitative analysis software. Study team members conducted interviews until thematic saturation was achieved.

### Analysis

Interview transcripts were analyzed using an iterative thematic analysis approach [14]. A coding team, comprised of 4 authors, read all the transcripts and developed preliminary codes. A few selected transcripts were coded by 2 research team members to ensure consistency in coding and to group codes into larger thematic classifications. One team member coded all the transcripts. Participant quotes were lightly edited for clarity.

Descriptive statistics, including frequencies, medians, and IQR, were calculated to summarize quantitative data from questionnaires and structured interview questions. Due to the small size of our sample, nonparametric Wilcoxon signed-rank tests were performed to compare clinicians’ recommendations for ED presentation without wearable device data to their recommendations after viewing wearable device data for each vignette. An α level of .05 was used as a cutoff for statistical significance. Analyses were performed in Python version 3.10 (Python Software Foundation).

### Ethical Considerations

This study was conducted at a National Cancer Institute–Designated Comprehensive Cancer Center. The institutional review board of the University of Pittsburgh approved all study activities (STUDY23120104). Informed consent was obtained from all individual participants included in the study. Participant data were identified only by anonymized study ID numbers and stored in secure locations. Data collection was conducted between March 2024 and June 2024. Participants received US $100 in compensation for their participation.

## Results

### Participant Characteristics

Participants included physicians (n=4), physician assistants (n=5), nurses (n=3), and a certified registered nurse practitioner (n=1) who had a mean age of 42 (SD 12.60; range 25‐61) years, were primarily White (12/13, 92%), and had been treating patients with cancer for an average of 9.7 (SD 6.42) years with a range from 5 months to 22 years.

### Survey

**Figure 1.**
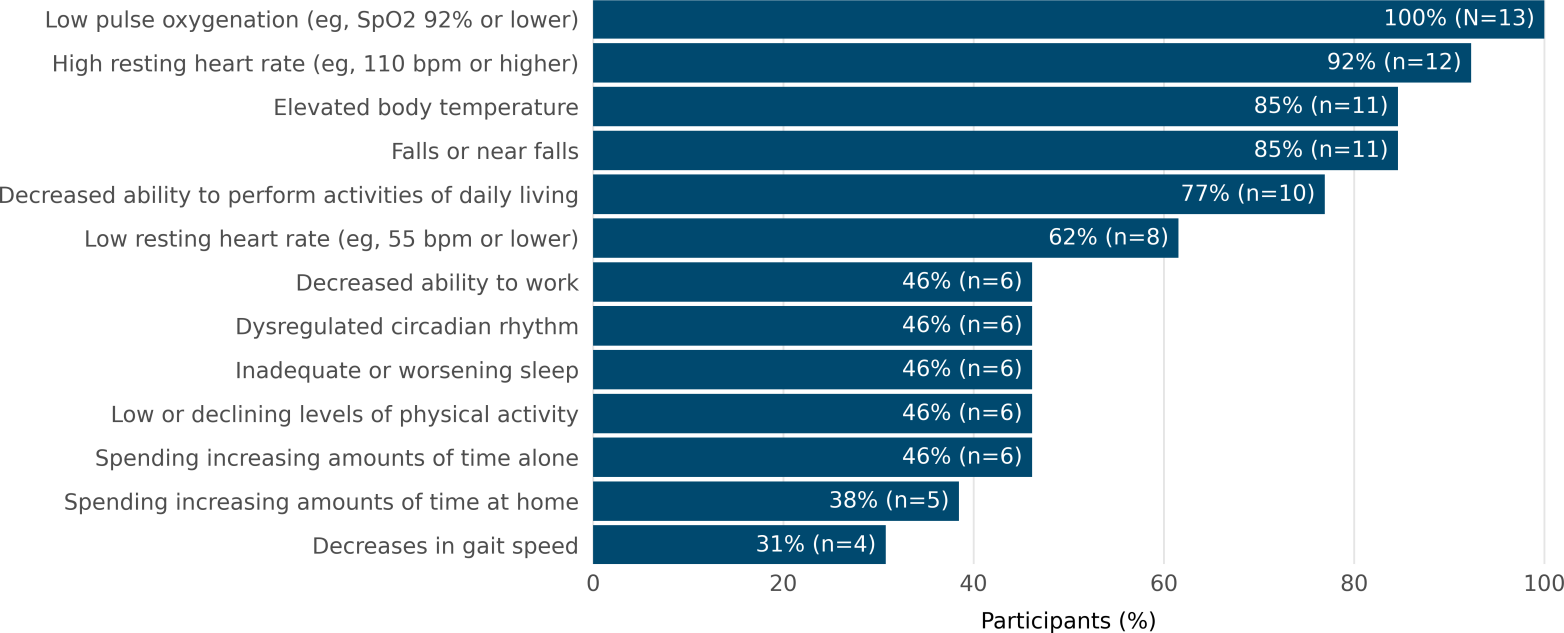
Number of participants who indicated they would want to know the following information about their patients.

**Figure 2.**
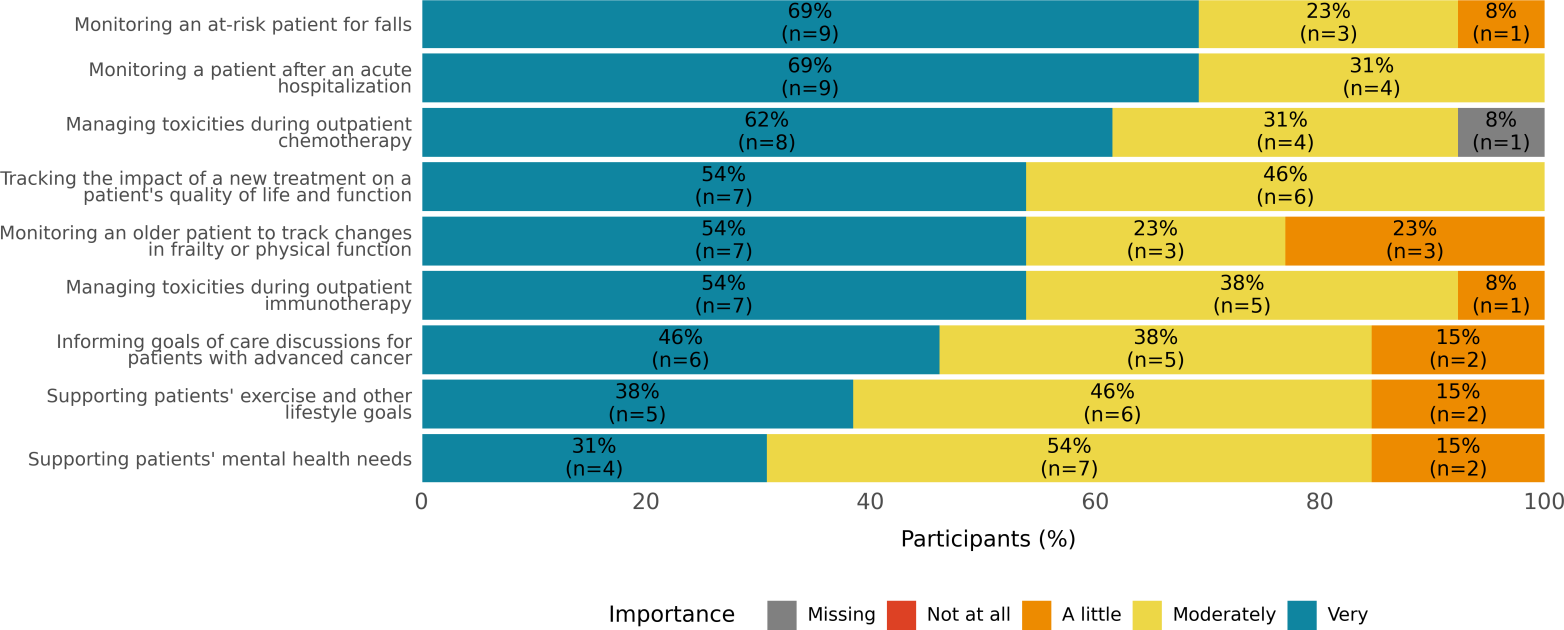
Potential clinical scenarios in which wearable devices might be valuable.

**Figure 3.**
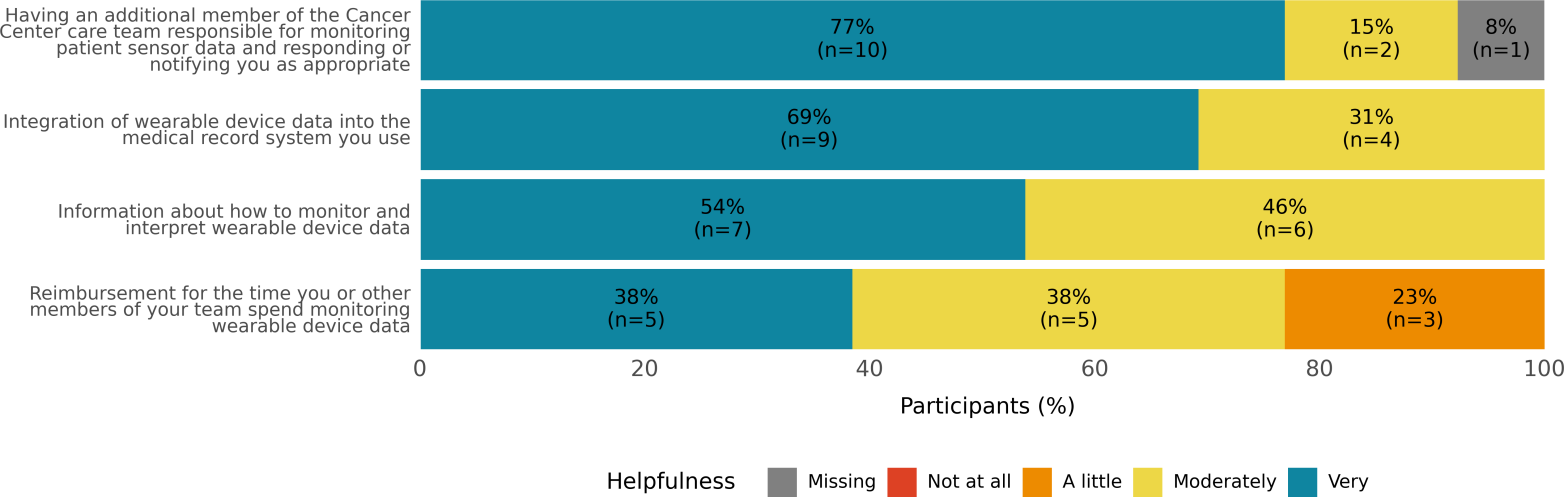
Perceived helpfulness of various supports for integrating wearable device data in the clinic.

The majority of providers (11/13, 85%) indicated that they wanted to know if their patients experienced low pulse oxygenation, high resting heart rate, elevated body temperature, and falls or near falls (Figure 1). All providers considered wearable device data to be moderately to very important when monitoring a patient after an acute hospitalization, managing toxicities during outpatient chemotherapy, and tracking the impact of a new treatment on a patient’s quality of life and function (Figure 2). When asked what would allow them to integrate wearable device data into their care, all providers responded that information about how to monitor and interpret wearable device data; having an additional member to monitor, respond to, and notify the team as appropriate; and integration of the wearable device data into the medical record system would be moderately to very helpful (Figure 3).

### Themes

During the semistructured interviews, four primary themes related to the benefits and barriers of integrating wearable device data into care were discussed: (1) corroborating reports, (2) identifying new issues, (3) coordinating care, and (4) patient-provider communication.

#### Theme 1: Corroborating Reports

Providers discussed how objective wearable device data could provide oncology clinics with a better understanding of patients’ concerns and symptoms between clinic visits.

*It would be nice if somebody’s calling me with questions, and I can just pull it up and see what’s really going on...It’s easier for people to kind of not understand the full grasp of things when they’re just talking. When you could just actually see the graph, it would probably be nice. But, also, I think it’s important for the providers to see it, because whenever they come in and they’re being seen by the providers, it would be good for them to be able to quantify what’s going on between visits*.

[P014, RN]

In particular, several providers mentioned that wearable device data could be used to help resolve conflicting reports they receive from patients and their support persons.

*Especially if maybe I was getting one story from the patient and a different story from their caregiver, or I was surprised by what I was hearing*.

[P007, MD]

*Sometimes I feel like patients don’t want to be dose reduced and they’ll lie. And then I try to usually get the family to rat them out like, “Okay, so are they really grocery shopping and cutting the lawn?” They’re like, “No, they're sitting on the couch all day.”*

[P003, MD]

#### Theme 2: Identifying New Issues

Some providers discussed the benefits of having wearable device data for identifying new issues that patients may not be reporting. One provider gave an example of a common scenario in which symptoms are typically experienced early in the chemotherapy cycle, but patients do not have another office visit for several weeks. This can lead to symptoms not being detected by the clinic or reported by the patient.

*But I feel like that would be really helpful, because when we see the patients for chemotherapy, we’re generally seeing them at the beginning of a cycle, right? And I feel like we have these people who rally and come in the office and look great because it’s been 3 weeks or 4 weeks, or something since their last chemo, and sometimes the data that we would have to kind of say, “Hey, you know, we might have to dose reduce you, because this whole first 10 day span, here was what was happening from the data that we got back, even if you're not telling us that.” So, I feel like that would be a valuable tool*.

[P016, PA]

#### Theme 3: Coordinating Care

Oncology patients often have a number of doctors with whom they must coordinate their care. A few providers additionally discussed how integrated wearable device data could improve patient care coordination across various clinicians and specialties.

*[T]here’s a lot of collaboration between teams. And I think, sometimes the timing of different therapies, and how people are responding, can kind of be indicative. So potentially in multi-disciplinary collaboration between, like, “Hey is a patient, you know, how do we know that they’re recovered from their chemo to get surgery? How do we know that they’re recovered from their surgery to get chemo? How do we know that they're not having these toxicities from radiation that would make us prevent them from going on?” So, I think there’d be a role to kind of integrate it from a medical oncology perspective, but also kind of have interplay between the groups, to kind of look at what the best avenue of approach of treatment might be. And that’s kind of outside of just, individual patient and clinician reporting, and that kind of thing. Just getting other teams involved, utilizing the data*.

[P016, PA]

#### Theme 4: Patient-Provider Communication

Finally, when discussing whether patients should be notified about significant changes in their wearable device data, most providers thought it would be beneficial for promoting patient advocacy.

*Yeah, I think their kind of awareness can only help the situation, even if it’s having a family member call in and say, “Yeah, we did notice this, this pulse ox was really low,” or “Yeah, she is having more trouble.” Even if they make the contact and it’s not necessarily just automatic from the data. If the patients alerted too, I think advocacy for themselves would be helpful*.

[P008, PA]

*Yes, so if they...became febrile or, their pulse ox was low, I would want them to be alerted. That way they could either call us if we weren’t getting back to them, or assess how they’re feeling. If your temp’s 100.0 but you feel okay, that’s not necessarily something we need to do anything about. But [it] would allow them, I think, to say, “Hey, this was my temp, but I felt fine,” or the opposite. And I think that would be helpful to correlate those two things together*.

[P015, PA]

However, several providers raised concerns about how these notifications would be triggered and suggested that careful wording should be used to prevent increasing patient anxiety.

*That’s a little bit harder, because I think it’s like, what does that mean? Right? What if it’s natural variation for them. I don’t want to alarm them. I don’t want them to think that there’s something critically wrong with them, if they otherwise feel fine, right. If your heart rate bounced up a little bit, but then it comes back down again. They probably already know if they’re not sleeping well, right. They probably already know if they feel bad. If it’s something that’s like a critical alert, then yes. Right. But that’s also a little bit nuanced. What’s critical? You have to set a value? But I don’t know that they need feedback on every little variation*.

[P005, MD]

*I feel like, in a sense, that could be good. But then, in another sense, it could just stress the patient out further if it’s a weekend, and somebody’s not getting back to them ASAP that might send them into a spiral*.

[P004, RN]

### Vignettes

Results related to the wearable device data vignettes are presented in Table 1. Across all 3 vignettes, the median difference in the likelihood of recommending ED presentation before and after viewing wearable device data was 0 (Q1 range: −0.5 to 0; Q3 range: 0 to 0.875). There were no statistically significant differences in the likelihood of recommending ED presentation before and after viewing wearable device data for any vignette (all *P*>.25). All clinicians reported that the wearable device data impacted their decision-making or confidence, and clinicians generally rated the wearable device data as helpful.

**Table 1.** Vignette responses (N=13).

Metric	Vignette 1	Vignette 2	Vignette 3
Likelihood of recommending ED^a^^,b^, median (IQR)			
Without wearable data	3 (3 to 5)	9 (8 to 9.5)	2 (0.75 to 2.625)
With wearable data	5.25 (2.875 to 6.25)	9 (9 to 10)	2 (1 to 2.5)
Difference^c^	0 (−0.5 to 0.875)^d^	0 (0 to 0)^e^	0 (−0.5 to 0)^f^
Change in recommendation with wearable data, n (%)			
More likely (difference>0)	4 (31)	3 (23)	2 (15)
No change (difference=0)	4 (31)	10 (77)	6 (46)
Less likely (difference<0)	4 (31)	0 (0)	4 (31)
Missing	1 (8)	0 (0)	1 (8)
Wearable data impacted decision making or confidence, n (%)	10 (77)	10 (77)	9 (69)
Helpfulness of wearable data^b^, median (IQR)	7 (4 to 8)	8 (5 to 9)	7 (6 to 8)

a ED: emergency department.

b Rating on 0‐10 scale.

c Wilcoxon signed-rank test (W=test statistic; *P*=*P* value; *r*=effect size).

d Vignette 1: W=13.5, *P*=.64, *r*=0.12.

e Vignette 2: W=0.0, *P*=.25, *r*=0.32.

f Vignette 3: W=7.0, *P*=.56, *r*=0.15.

## Discussion

### Principal Results

The goal of this mixed methods study was to examine oncology clinicians’ perspectives on integrating wearable device data into clinical decision-making and care. Overall, providers noted many potential benefits of collecting wearable device data during outpatient treatment for patients with cancer and sharing these data with clinical care teams. Survey responses helped pinpoint which wearable device data providers would most like to be notified about, with clinicians indicating that they were most interested in tracking vital sign metrics, followed by data related to falls and functioning, and then by data on sleep and activity. Clinicians thought that wearable device data might be especially useful for remotely monitoring patients at high-risk moments in their care trajectory, such as after an acute hospitalization or after starting a new outpatient treatment.

Themes from interviews highlight that wearable device data could serve several useful functions in the context of cancer care, including providing objective data to corroborate, clarify, or reconcile contradictory subjective reports; helping to identify new issues that emerge when the patient is outside of the clinic; and providing objective baseline and historical data that might serve as a foundation for the coordination of multidisciplinary cancer care. Clinicians further noted both positive and negative implications for patient-provider communication. While wearable device data could help patients with cancer take a more active role in monitoring and managing their health, clinicians also noted that the patient-facing interfaces and notifications must be thoughtfully implemented to prevent undue patient anxiety and distress. Providing information on how to interpret wearable device data, adding an additional team member to monitor notifications about changes in these data, and integrating this information into the electronic medical record were judged as being potentially moderately to very helpful for future clinical integration.

Clinicians’ responses to vignettes in which clinical scenarios were presented with or without visualizations of wearable device data collected from patients further demonstrated the potential clinical value of this technology, with clinicians rating the helpfulness of the data visualizations highly and noting that the data visualizations increased confidence in their clinical recommendations even though these recommendations were not changed by the data. Even if recommendations do not shift, increased clinician confidence in decisions and recommendations may be clinically significant, with benefits that could include enhanced patient trust in clinicians, reduced time spent on clinical decision-making, and decreased clinician burnout.

### Comparison With Prior Work

These results are consistent with findings from previous studies in which noncancer physicians reported that wearable device data could improve communication and symptom management [8,9]. Similarly, oncology physicians were also concerned about whether alerts might cause undue anxiety for their patients [10]. Beyond the previously documented benefits and disadvantages, providers in this study indicated which specific wearable device data would be most relevant to their clinical needs and identified the support necessary to integrate this information into their clinical workflow. Furthermore, although providers’ ED recommendations did not change significantly before and after viewing the wearable device data, they reported increases in confidence comparable to those observed in the pediatric surgery vignette study that informed this aspect of the present study [13].

### Limitations

This study used multiple methods, including online surveys, semistructured interviews, and clinical vignettes augmented with visualizations of oncology research patients’ wearable device data, to begin to probe cancer clinicians’ perspectives on wearable devices in cancer care. Limitations of this study include the small sample of clinicians from a single academic cancer center who were interested in participating in a study about wearable devices and who may be favorably biased toward digital health. The small sample also constrained our ability to examine different perspectives across disciplines (eg, among physicians vs nurses), disease specialties, or different clinics or care facilities, and exploration of how perspectives differ across these roles and settings should be a focus of future research. The survey and interview questions were not adapted from validated instruments or pilot-tested and suffered from various conceptual and methodological weaknesses. Additionally, the vignettes were based on a convenience selection of available wearable device data from our research group and therefore focused on patients with stage IV cancer who used a Fitbit Inspire device [7]. Findings may be different if vignettes focused on patients with early-stage cancer undergoing curative treatment or if data from a different wearable device were presented. Because these vignettes were selected retrospectively from a research study, they may not reflect real-world clinical scenarios where this information would be best applied.

Despite these limitations, this work contributes to a growing body of literature on the value of wearable and other mobile sensors in clinical oncology and highlights cautious enthusiasm among clinicians about how these data could inform and improve cancer care.

### Future Directions

Findings suggest that oncology providers recognize the clinical value of integrating wearable device data into their workflow and that such integration may enhance their confidence in decision-making. More work is needed to identify the threshold at which wearable device data signals increasing risk as well as the best way to communicate this risk to providers and patients to avoid straining clinical resources or increasing patient anxiety or clinician alert fatigue. Research has suggested that early comprehensive training and additional support within the care team can mitigate burnout, but further investigation is needed into how this will be structured within oncology clinics to minimize cost and burden [15]. Additionally, future studies should examine how providing clinicians with access to wearable device data impacts clinical decision-making, patient-provider communication, and patient outcomes.

Future studies exploring wearable device data integration into oncology care should also acknowledge issues related to cost and access. Disparities exist along socioeconomic lines as well as between rural and urban areas, which, if left unchecked, could result in further inequity and widen the digital divide. Those with lower incomes, less educational achievement, and those living in rural areas are less likely to own wearable devices [16,17]. Simply supplying these devices to patients may not be sufficient either, as previous work has demonstrated that demographic and clinical factors, such as non-White race and cognitive impairments, have been linked to lower adherence to wearable devices [4]. Further investigation is needed into ways to improve digital health literacy in patients with cancer, in addition to improving access, to ensure wearable device data integration does not continue to advantage only certain populations.

In conclusion, oncology providers view patient-generated wearable device data as potentially valuable in the cancer care setting. They indicated that access to this information could improve communication between providers and patients, as well as between collaborating clinicians. Displaying wearable data alongside symptom data may also be helpful in contextualizing patient and caregiver reports. While the wearable device data visualizations did not change hypothetical clinical recommendations, providers reported that the data increased confidence in their decisions. As wearable devices become more common, it becomes increasingly important to have systems in place that allow these new real-world data streams to be integrated into the oncology clinic workflow.

## Supplementary material

10.2196/86412 Multimedia Appendix 1 Interviews for remote oncology symptom assessment 2 provider questionnaire.

10.2196/86412 Multimedia Appendix 2 Interviews for remote oncology symptom assessment 2 provider interview guide.

## Abbreviations

ED: emergency department
