# Supplementary material for: Clinician Perspectives on Integrating Mobile Sensor Data Into Cancer Care: Mixed Methods Study
Source: JMIR Cancer. 2026 May 8;12:e86412. doi: 10.2196/86412 (PMC13155500; doi:10.2196/86412)
Supplement: Multimedia Appendix 1 [file cancer-v12-e86412-s001.pdf]

Provider Questionnaire

Please complete the survey below.

Thank you!

Date/Time Completed

____

---

1. What sex were you assigned at birth?

◯ Male
◯ Female

---

2. How do you describe yourself?

◯ Male
◯ Female
◯ Transgender
◯ Other

If Other, please specify:

____

---

3. What is your date of birth?

____
(MM-DD-YYYY)

Age

____

---

4. Do you consider yourself to be of Latin or Hispanic origin?

◯ Yes
◯ No

---

5. What is your race/ethnicity? (select all that apply)

☐ White/Caucasian
☐ Black/African American
☐ American Indian or Alaska Native
☐ Asian
☐ Native Hawaiian or Other Pacific Islander
☐ Other

If Other race/ethnicity, please specify:

____

---

6. What is your role in treating oncology patients?

◯ Physician
◯ Physician Assistant
◯ Nurse
◯ Other

If Other role, please specify:

____

7. Approximately how long have you been treating patients with cancer and/or patients receiving chemotherapy?

____

For the next set of questions, think about outpatients receiving chemotherapy under your care.

Which of the following symptoms would you want to know about if your patient experienced them at home in real time? (check all that apply)

- ☐ Decreased appetite
- ☐ Nausea
- ☐ Vomiting
- ☐ Constipation
- ☐ Loose or watery stools (diarrhea/diarrhoea)
- ☐ Pain in the abdomen (belly area)
- ☐ Shortness of breath
- ☐ Rash
- ☐ Numbness or tingling in your hands or feet
- ☐ Dizziness
- ☐ Insomnia (including difficulty falling asleep, staying asleep, or waking up early)
- ☐ Fatigue, tiredness, or lack of energy
- ☐ Anxiety
- ☐ Sad or unhappy feelings
- ☐ Problems with concentration
- ☐ Problems with memory
- ☐ Pain
- ☐ Urinary Problems
- ☐ Sexual Health and Intimacy Problems
- ☐ Weight loss

When would you want to be notified about decreased appetite? (check all that apply)

- ☐ Anytime your patient rates it as very severe
- ☐ Anytime your patient rates it as severe
- ☐ Anytime your patient rates it as moderate
- ☐ Anytime your patient rates it as mild
- ☐ Anytime your patient reports it for the first time
- ☐ Anytime your patient reports that it is worsening in severity
- ☐ Anytime your patient reports it multiple days in a row

When would you want to be notified about nausea? (check all that apply)

- ☐ Anytime your patient rates it as very severe
- ☐ Anytime your patient rates it as severe
- ☐ Anytime your patient rates it as moderate
- ☐ Anytime your patient rates it as mild
- ☐ Anytime your patient reports it for the first time
- ☐ Anytime your patient reports that it is worsening in severity
- ☐ Anytime your patient reports it multiple days in a row

When would you want to be notified about vomiting? (check all that apply)

- ☐ Anytime your patient rates it as almost constantly
- ☐ Anytime your patient rates it as frequently
- ☐ Anytime your patient rates it as occasionally
- ☐ Anytime your patient rates it as rarely
- ☐ Anytime your patient reports it for the first time
- ☐ Anytime your patient reports that it is worsening in severity
- ☐ Anytime your patient reports it multiple days in a row

When would you want to be notified about constipation? (check all that apply)

- ☐ Anytime your patient rates it as very severe
- ☐ Anytime your patient rates it as severe
- ☐ Anytime your patient rates it as moderate
- ☐ Anytime your patient rates it as mild
- ☐ Anytime your patient reports it for the first time
- ☐ Anytime your patient reports that it is worsening in severity
- ☐ Anytime your patient reports it multiple days in a row

When would you want to be notified about loose or watery stool (diarrhea/diarrhoea)? (check all that apply)

- ☐ Anytime your patient rates it as almost constantly
- ☐ Anytime your patient rates it as frequently
- ☐ Anytime your patient rates it as occasionally
- ☐ Anytime your patient rates it as rarely
- ☐ Anytime your patient reports it for the first time
- ☐ Anytime your patient reports that it is worsening in severity
- ☐ Anytime your patient reports it multiple days in a row

When would you want to be notified about pain in the abdomen (belly area)? (check all that apply)

- ☐ Anytime your patient rates it as very severe
- ☐ Anytime your patient rates it as severe
- ☐ Anytime your patient rates it as moderate
- ☐ Anytime your patient rates it as mild
- ☐ Anytime your patient reports it for the first time
- ☐ Anytime your patient reports that it is worsening in severity
- ☐ Anytime your patient reports it multiple days in a row

When would you want to be notified about shortness of breath? (check all that apply)

- ☐ Anytime your patient rates it as very severe
- ☐ Anytime your patient rates it as severe
- ☐ Anytime your patient rates it as moderate
- ☐ Anytime your patient rates it as mild
- ☐ Anytime your patient reports it for the first time
- ☐ Anytime your patient reports that it is worsening in severity
- ☐ Anytime your patient reports it multiple days in a row

When would you want to be notified about a rash? (check all that apply)

- ☐ Anytime your patient rates it as very severe
- ☐ Anytime your patient rates it as severe
- ☐ Anytime your patient rates it as moderate
- ☐ Anytime your patient rates it as mild
- ☐ Anytime your patient reports it for the first time
- ☐ Anytime your patient reports that it is worsening in severity
- ☐ Anytime your patient reports it multiple days in a row

When would you want to be notified about numbness or tingling in your hands or feet? (check all that apply)

- ☐ Anytime your patient rates it as very severe
- ☐ Anytime your patient rates it as severe
- ☐ Anytime your patient rates it as moderate
- ☐ Anytime your patient rates it as mild
- ☐ Anytime your patient reports it for the first time
- ☐ Anytime your patient reports that it is worsening in severity
- ☐ Anytime your patient reports it multiple days in a row

When would you want to be notified about dizziness? (check all that apply)

- ☐ Anytime your patient rates it as very severe
- ☐ Anytime your patient rates it as severe
- ☐ Anytime your patient rates it as moderate
- ☐ Anytime your patient rates it as mild
- ☐ Anytime your patient reports it for the first time
- ☐ Anytime your patient reports that it is worsening in severity
- ☐ Anytime your patient reports it multiple days in a row

When would you want to be notified about insomnia (including difficulty falling asleep, staying asleep, or waking up early)? (check all that apply)

- ☐ Anytime your patient rates it as very severe
- ☐ Anytime your patient rates it as severe
- ☐ Anytime your patient rates it as moderate
- ☐ Anytime your patient rates it as mild
- ☐ Anytime your patient reports it for the first time
- ☐ Anytime your patient reports that it is worsening in severity
- ☐ Anytime your patient reports it multiple days in a row

When would you want to be notified about fatigue, tiredness, or lack of energy? (check all that apply)

- ☐ Anytime your patient rates it as very severe
- ☐ Anytime your patient rates it as severe
- ☐ Anytime your patient rates it as moderate
- ☐ Anytime your patient rates it as mild
- ☐ Anytime your patient reports it for the first time
- ☐ Anytime your patient reports that it is worsening in severity
- ☐ Anytime your patient reports it multiple days in a row

When would you want to be notified about anxiety? (check all that apply)

- ☐ Anytime your patient rates it as very severe
- ☐ Anytime your patient rates it as severe
- ☐ Anytime your patient rates it as moderate
- ☐ Anytime your patient rates it as mild
- ☐ Anytime your patient reports it for the first time
- ☐ Anytime your patient reports that it is worsening in severity
- ☐ Anytime your patient reports it multiple days in a row

When would you want to be notified about sad or unhappy feelings? (check all that apply)

- ☐ Anytime your patient rates it as very severe
- ☐ Anytime your patient rates it as severe
- ☐ Anytime your patient rates it as moderate
- ☐ Anytime your patient rates it as mild
- ☐ Anytime your patient reports it for the first time
- ☐ Anytime your patient reports that it is worsening in severity
- ☐ Anytime your patient reports it multiple days in a row

When would you want to be notified about problems with concentration? (check all that apply)

- ☐ Anytime your patient rates it as very severe
- ☐ Anytime your patient rates it as severe
- ☐ Anytime your patient rates it as moderate
- ☐ Anytime your patient rates it as mild
- ☐ Anytime your patient reports it for the first time
- ☐ Anytime your patient reports that it is worsening in severity
- ☐ Anytime your patient reports it multiple days in a row

When would you want to be notified about problems with memory? (check all that apply)

- ☐ Anytime your patient rates it as very severe
- ☐ Anytime your patient rates it as severe
- ☐ Anytime your patient rates it as moderate
- ☐ Anytime your patient rates it as mild
- ☐ Anytime your patient reports it for the first time
- ☐ Anytime your patient reports that it is worsening in severity
- ☐ Anytime your patient reports it multiple days in a row

When would you want to be notified about pain? (check all that apply)

- ☐ Anytime your patient rates it as very severe
- ☐ Anytime your patient rates it as severe
- ☐ Anytime your patient rates it as moderate
- ☐ Anytime your patient rates it as mild
- ☐ Anytime your patient reports it for the first time
- ☐ Anytime your patient reports that it is worsening in severity
- ☐ Anytime your patient reports it multiple days in a row

When would you want to be notified about urinary problems? (check all that apply)

- ☐ Anytime your patient rates it as very severe
- ☐ Anytime your patient rates it as severe
- ☐ Anytime your patient rates it as moderate
- ☐ Anytime your patient rates it as mild
- ☐ Anytime your patient reports it for the first time
- ☐ Anytime your patient reports that it is worsening in severity
- ☐ Anytime your patient reports it multiple weeks in a row

When would you want to be notified about sexual health and intimacy problems? (check all that apply)

- ☐ Anytime your patient rates it as very severe
- ☐ Anytime your patient rates it as severe
- ☐ Anytime your patient rates it as moderate
- ☐ Anytime your patient rates it as mild
- ☐ Anytime your patient reports it for the first time
- ☐ Anytime your patient reports that it is worsening in severity
- ☐ Anytime your patient reports it multiple weeks in a row

At what threshold (lbs lost per week) would you like to be notified about weight loss?

____

Any other symptoms not listed? At what severity would you like to be notified of these symptoms? Any other contextual information that may be important to consider when setting thresholds for notifications?

Which of the following information would you want to know about if your patient experienced them? (check all that apply)

☐ Low or declining levels of physical activity
☐ Inadequate or worsening sleep
☐ Dysregulated circadian rhythm (eg frequently being awake overnight and/or asleep during the day, with no consistent bedtime or waketime)
☐ High resting heart rate (eg 110 bpm or higher)
☐ Low resting heart rate (eg 55 bpm or lower)
☐ Elevated body temperature
☐ Low pulse oxygenation (eg SpO2 92% or lower)
☐ Decreased ability to perform activities of daily living
☐ Decreased ability to work
☐ Decreases in gait speed
☐ Falls or near falls
☐ Spending increasing amounts of time at home
☐ Spending increasing amounts of time alone

Any other data that might be available from a wearable device that you would want to know about?

____

In which of the following scenarios do you think data from wearable devices might be valuable:

Monitoring a patient after an acute hospitalization
◯ Not at all important
◯ A little important
◯ Moderately important
◯ Very important

Comments: ______________________________

Monitoring an older patient to track changes in frailty or physical function
◯ Not at all important
◯ A little important
◯ Moderately important
◯ Very important

Comments: ______________________________

Managing toxicities during outpatient chemotherapy
◯ Not at all important
◯ A little important
◯ Moderately important
◯ Very important

Comments: ______________________________

Managing toxicities during outpatient immunotherapy
◯ Not at all important
◯ A little important
◯ Moderately important
◯ Very important

Comments: ______________________________

Tracking the impact of a new treatment on a patient's quality of life and function

- ◯ Not at all important
- ◯ A little important
- ◯ Moderately important
- ◯ Very important

Comments: ________________________________

Informing goals of care discussions for advanced cancer patients

- ◯ Not at all important
- ◯ A little important
- ◯ Moderately important
- ◯ Very important

Comments: ________________________________

Monitoring an at-risk patient for falls

- ◯ Not at all important
- ◯ A little important
- ◯ Moderately important
- ◯ Very important

Comments: ________________________________

Supporting patients' exercise and other lifestyle goals

- ◯ Not at all important
- ◯ A little important
- ◯ Moderately important
- ◯ Very important

Comments: ________________________________

Supporting patients' mental health needs

- ◯ Not at all important
- ◯ A little important
- ◯ Moderately important
- ◯ Very important

Comments: ________________________________

Other: ________________________________

How helpful would the following be in allowing you to integrate wearable device data into the care you provide?

Information about how to monitor and interpret wearable device data

- ◯ Not at all helpful
- ◯ A little helpful
- ◯ Moderately helpful
- ◯ Very helpful

Reimbursement for the time you or other members of your team spend monitoring wearable device data

- ◯ Not at all helpful
- ◯ A little helpful
- ◯ Moderately helpful
- ◯ Very helpful

Having an additional member of the Cancer Center care team responsible for monitoring patient sensor data and responding or notifying you as appropriate	◯ Not at all helpful<br>◯ A little helpful<br>◯ Moderately helpful<br>◯ Very helpful
Integration of wearable device data into the medical record system you use	◯ Not at all helpful<br>◯ A little helpful<br>◯ Moderately helpful<br>◯ Very helpful
Other:	__________________________________
