# Supplementary material for: Clinician Perspectives on Integrating Mobile Sensor Data Into Cancer Care: Mixed Methods Study
Source: JMIR Cancer. 2026 May 8;12:e86412. doi: 10.2196/86412 (PMC13155500; doi:10.2196/86412)
Supplement: Multimedia Appendix 2 [file cancer-v12-e86412-s002.pdf]

Provider Interview Guide

"Our team has been developing a remote monitoring system where patients wear a Fitbit and report their daily symptoms on their phone. We've reached 200 participants and are putting together predictions on how those patients might feel the following day. We're also trying to share these symptoms and sensor data with providers, but we need your help in determining the easiest way to integrate this information into the clinical workflow."

- First, we would like you to share your experiences providing care to oncology patients:
  - How long have you been working as an oncology clinician, and what kinds of cancer patients do you typically work with?
  - Can you share what a clinical day looks like for you? You can share anything from your daily routine (if any), how many patients you see on a given day, how do you handle patient follow-ups, etc.
- How interested would you be in having access to data related to [anything endorsed in questionnaire]?
- Which person or people on your team do you think would be the best person to have access to and review these data?
- How would you want to be notified about [anything endorsed in the questionnaire] that happens at home? (e.g., alert, email, notify nurse or other member of the team, other)
- Is there other information you would want to collect before taking clinical action?
- Would you want your patient to be notified about [anything endorsed in the questionnaire]? (MyUPMC, phone, text, email, schedule clinic appointment, video visit, other)
- If there was a dashboard to view these data, how likely would you be to view it:
  - Together with a patient at their clinic visit (not at all; somewhat; very)
  - When a patient calls to report an issue or concern (not at all; somewhat; very)
  - When a patient is hospitalized unexpectedly (not at all; somewhat; very)
  - Prior to seeing a patient for a scheduled visit (not at all; somewhat; very)
  - Are there any other situations where you would like to view a dashboard with these data?
- How interested would you be in having access to predictions based on wearable data that:
  - Your patient is at risk of a hospitalization
  - Your patient is at risk of a fall
  - Your patient is at risk of a chemotherapy dose reduction
  - Are there any other predictions based on wearable data that you would be interested in having access to?

“Next, we’ll show you actual wearable device data from three ROSA participants, along with clinical information and scenarios. We’re interested to know whether and how the wearable device data might impact your clinical decision-making and understanding of the patient as well as what you like and dislike about each visualization.”

Clinical Vignette #1: Phone call

Patient is a 75 y/o male with pancreatic cancer, underwent a surgical procedure 3 months ago and currently receiving FOLFIRINOX (last cycle 2 days ago). Reports worsening abdominal pain, nausea, vomiting, anorexia, and weakness over the last few days. No diarrhea, headache, shortness of breath, or other symptoms.

Prior to seeing wearable device data:

- What would you recommend?
  - 1 = go to the ED immediately
  - 2 = prescribe medication with outpatient follow-up
  - 3 = outpatient follow-up only
  - 4 = provide reassurance and instructions for when to call back
  - 5 = other
- On a scale from 0-10, how likely are you to recommend the patient to present to the ED immediately?
- Is there anything else you’d want to know to inform your recommendation?

[heat maps of heart rate data and maximum 5-minute walking cadence]

After seeing wearable device data:

- On a scale from 0-10, how likely are you to recommend the patient to present to the ED immediately?
- Did the wearable device data have any impact on your decision-making or your confidence in your decision?
- On a scale from 0-10, how helpful was the wearable device data to your understanding of the situation?
- Other comments?

Clinical Vignette #2: Phone call

Patient is a 58 y/o male with Stage IV pancreatic adenocarcinoma with liver metastases currently on chemotherapy (Fluorouracil + Leucovorin + Irinotecan, most recent treatment 10 days ago). He reports that over the past 4-5 days, he has been experiencing dizziness as well as intermittent fever and chills with some abdominal pain. Denies headaches, chest pain, shortness of breath, other symptoms.

Prior to seeing wearable device data:

- What would you recommend?
    - 1 = go to the ED immediately
    - 2 = prescribe medication with outpatient follow-up
    - 3 = outpatient follow-up only
    - 4 = provide reassurance and instructions for when to call back
    - 5 = other
- On a scale from 0-10, how likely are you to recommend the patient to present to the ED immediately?
- Is there anything else you'd want to know to inform your recommendation?

[line graphs of step count and average gait speed and heart rate with asterisks denoting significant change]

After seeing wearable device data:

- On a scale from 0-10, how likely are you to recommend the patient to present to the ED immediately?
- Did the wearable device data have any impact on your decision-making or your confidence in your decision?
- On a scale from 0-10, how helpful was the wearable device data to your understanding of the situation?
- Other comments?

Clinical Vignette #3: Scheduled visit

Patient is a 56 y/o female with Stage IV breast cancer with liver and bone metastases. She was receiving Carboplatin and Abraxane. She was admitted for 10 days for cellulitis and pleural effusion. She began Enhertu treatment three days after being released. Fifteen days after she was released and twelve days after her change in treatment, she reported worsening symptoms, including fatigue, nausea, and decreased appetite.

Prior to seeing wearable device data:

- What would you recommend? (indicate all that apply)
    - 1 = hold or reduce treatment
    - 2 = prescribe medication(s) to manage symptoms
    - 3 = consider alternative treatment
    - 4 = goals of care conversation
    - 5 = provide reassurance and instructions for when to call the office
    - 6 = other

- On a scale from 0-10, how likely are you to recommend the patient to present to the ED immediately?
- Is there anything else you'd want to know to inform your recommendation?

[line graphs of step count, hours of sleep, and average gait speed and heart rate]

After seeing wearable device data:

- On a scale from 0-10, how likely are you to recommend the patient to present to the ED immediately?
- Did the wearable device data have any impact on your decision-making or your confidence in your decision?
- On a scale from 0-10, how helpful was the wearable device data to your understanding of the situation?
- Other comments?
